# A dual program for CRP-mediated regulation in bacterial alarmone (p)ppGpp

**DOI:** 10.1128/mbio.02430-24

**Published:** 2024-10-04

**Authors:** Li Zhao, Shi-Yu Zhou, Yu Fu, Jin-Long Shen, Bin-Cheng Yin, Di You, Bang-Ce Ye

**Affiliations:** 1 Lab of Biosystems and Microanalysis, State Key Laboratory of Bioreactor Engineering, East China University of Science and Technology, Shanghai, China; 2 Institute of Engineering Biology and Health, Collaborative Innovation Center of Yangtze River Delta Region Green Pharmaceuticals, College of Pharmaceutical Sciences, Zhejiang University of Technology, Hangzhou, Zhejiang, China; University of Washington School of Medicine, Seattle, Washington, USA; Loyola University Chicago–Health Sciences Campus, Maywood, Illinois, USA

**Keywords:** cAMP-CRP, (p)ppGpp, protein acetylation, transcriptional regulation, translational regulation

## Abstract

Gene expression and proper downstream cellular functions upon facing environmental shifts depend on the combined and cooperative regulation of genetic networks. Here, we identified cAMP receptor protein (CRP) as a master regulator of (p)ppGpp (guanosine tetra- and penta-phosphate) homeostasis. Via CRP-mediated direct transcriptional regulation of the (p)ppGpp synthetase/hydrolase RelA and SpoT, cAMP-CRP stimulates pervasive accumulation of (p)ppGpp under glucose-limiting conditions. Notably, CRP exerts a nonclassical property as a translational regulator through YfiQ-dependent acetylation of ribosome protein S1 at K247, which further enhances the translation of RelA, SpoT, and CRP itself. From a synthetic biology perspective, this self-activating feedback loop for (p)ppGpp synthesis highlights the function of **C**RP-**m**ediated **d**ual **e**nhancement (CMDE) in controlling bacterial gene expression, which enables stable activation of genetic circuits. CMDE applied in synthetic circuits leads to a stable increase in *p*-coumaric acid, cinnamic acid, and pinosylvin production. Our findings showed that CRP-mediated dual circuits for (p)ppGpp regulation enable robust activation that could address bioproduction and other biotechnological needs.

**IMPORTANCE:**

Transcriptional–translational coordination is fundamental for rapid and efficient gene expression in most bacteria. Here, we uncovered the roles of cAMP-CRP in this process. We found that CRP distinctly increases RelA and SpoT transcription and translation, and that acetylation of S1 at K247 accelerates the self-activation of the leading CRP under glucose-limiting conditions. We further found that elevated (p)ppGpp significantly impedes the formation of the cAMP-CRP complex, an active form responsible for transcriptional activation. A model was created in which cAMP-CRP and (p)ppGpp cooperate to dynamically modulate the efficiency of transcriptional–translational coordination responses to stress. More broadly, productive activation in synthetic circuits was achieved through the application of **C**RP-**m**ediated **d**ual **e**nhancement (CMDE), promising to inspire new approaches for the development of cell-based biotechnologies.

## INTRODUCTION

Bacteria encounter numerous stresses in harsh environments. Therefore, fast and robust adaptive responses are essential for bacterial survival. Proper cellular function depends on the connections of genetic networks, including transcriptional regulation, translational regulation, and stress signaling pathways that rely on small nucleotide messengers (1, 2). The cAMP receptor protein (CRP) is a well-established transcription factor (TF) that regulates the transcription of over 250 genes by responding to changes in the intracellular cAMP level in *Escherichia coli* (3) and is often considered a prokaryotic protein model (4, 5). CRP is a homodimer, and the form of CRP-(cAMP)_2_ is responsible for transcriptional activation (6). cAMP is produced mainly when glucose is limited (5) and plays a major role in carbon catabolite repression (CCR) (5, 7, 8). Although CRP is a global regulator that controls transcription initiation from hundreds of promoters, *in vivo* transcriptional profiling has failed to identify most of the CRP-dependent regulatory effects due to the complexity of the regulatory network.

Another hotspot of genetic regulation orchestrating pleiotropic adaptations to nutritional starvation and stress is stringent response regulation, which relies on the alarmone (p)ppGpp (1, 9–12). Elevated (p)ppGpp levels initiate the stringent response, accompanied by significant cellular reprogramming, such as suppression of rRNA and tRNA synthesis, activation of stress-related genes, alterations in mRNA stability and translation, and optimization of the allocation of limited resources (10). The dynamic modulation of cellular (p)ppGpp levels is paramount for maintaining cellular homeostasis. During the stringent response, members of the RelA/SpoT homology (RSH)-type protein family are central to the metabolism of (p)ppGpp (13). The activity of RSH proteins is regulated by various mechanisms, and (p)ppGpp-inducing conditions include the presence of stalled ribosomes (14, 15), fatty acid starvation and carbon downshifts (16, 17), increased transcription of (p)ppGpp synthetases through cell wall stress stimuli (18, 19), and allosteric stimulation through (p)ppGpp itself (20, 21).

Despite rapid advances in research thus far, which has provided a partial understanding of the proposed mechanisms of signal transduction on the basis of (p)ppGpp and CRP (22, 23), our knowledge on these genetic networks is still fragmented. In this study, we present genetic assays for the identification of cAMP-CRP, which can modulate (p)ppGpp synthesis in *E. coli*. CRP is required for maintaining elevated (p)ppGpp levels in response to glucose starvation. We showed that CRP directly activates the transcription of RelA and SpoT. Through examining its known effects on YfiQ-catalyzed acetylation, we determined that S1 acetylation at K247 contributes to translational activation of RelA, SpoT, and CRP. Therefore, we propose that CRP functions as a master regulator of the intracellular concentration of (p)ppGpp through direct transcriptional regulation and indirect translational regulation. Importantly, (p)ppGpp competes directly with cAMP for binding to CRP, which in turn regulates CRP-mediated regulation. These results suggest that regulation by cAMP-CRP is important for adjusting the (p)ppGpp level and coordinating cellular metabolism in response to glucose availability. Moreover, a strategy functionally based on this CRP-mediated dual program can be used to effectively stabilize circuit function.

## RESULTS

### CRP influences intracellular (p)ppGpp levels via direct transcriptional activation of RelA and SpoT

**Fig 1:**
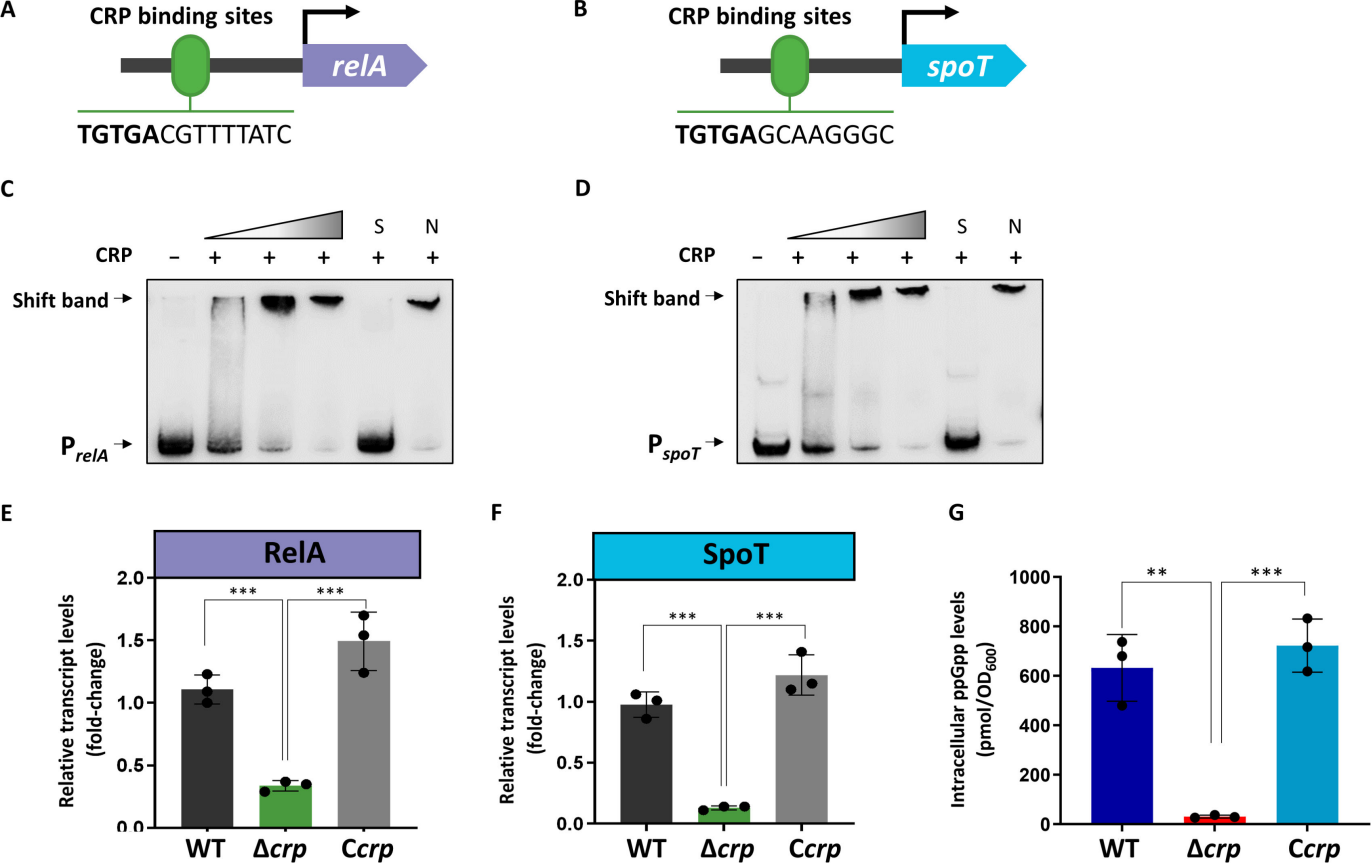
CRP influences intracellular (p)ppGpp levels via direct transcriptional activation of RelA and SpoT. (A) CRP binding sites in the *relA* promoter region of *E. coli*. (B) CRP binding sites in the *spoT* promoter region of *E. coli*. EMSAs of purified His-tagged CRP with the *relA* (C) and *spoT* (D) promoter regions. DNA probes (10 nM) were incubated with serial dilutions of purified CRP. EMSAs with a 200-fold excess of unlabeled specific probe (S) or nonspecific competitor DNA (sperm DNA) (N) were used as controls. The transcription levels of *relA* (E) and *spoT* (F) in the *E. coli* WT, Δ*crp,* and C*crp* strains grown until late exponential growth in liquid M9 minimal medium supplemented with 5 mM glucose as carbon resource. The mean value in the WT strain was set to 1.0 (arbitrary units). The fold change represents the expression level compared to the mean value in the WT strain. (G) Intracellular (p)ppGpp concentrations in cell extracts of the *E. coli* WT, Δ*crp,* and C*crp* strains grown in liquid M9 minimal medium supplemented with 5 mM glucose as carbon resource during late exponential growth. The (p)ppGpp concentrations of the samples were normalized to the OD_600_. The error bars show the standard deviations (SDs) of three independent experiments, and the mean values from independent experiments were used. An ordinary one-way analysis of variance (ANOVA) was performed to test the significance. **P < 0.01, ***P < 0.001.

CRP functions as a dimer in the form of a cAMP-CRP complex and regulates transcription initiation by binding to a DNA sequence containing 5′-TGTGA-3′, near or within the promoter regions (6, 24). Putative CRP binding sites were identified in the upstream regions of the *relA* and *spoT* genes in *E. coli* (Fig. 1A and B). To investigate whether CRP could directly bind to the upstream regions of *relA* and *spoT*, electrophoretic mobility shift assays (EMSAs) were performed. A 200-fold excess of unlabeled specific probes (S) and nonspecific competitor DNA (sperm DNA) (N) were used as controls. Figure 1C and D showed obvious shifts in the bands following incubation with the purified His-tag CRP in the presence of cAMP, suggesting that cAMP-CRP binds to the promoter regions of *relA* and *spoT*. We then examined whether CRP has a regulatory effect on *relA* and *spoT* using the Δ*crp* strain and *crp*-complemented strain (C*crp*), which were constructed as described previously (25). The transcription levels of *relA* and *spoT* in the *E. coli* wild-type (WT), Δ*crp*, and C*crp* strains were detected. As shown in Fig. 1E and F, compared with the WT strain, the *crp* deficiency caused a 70% decrease in *relA* transcription and a much more dramatic (10-fold) reduction in *spoT* transcription; these effects were reversed in the *crp*-complemented strain. Therefore, these results show that the cAMP-CRP complex positively regulates the transcription of the *relA* and *spoT* genes in *E. coli*.

In *E. coli*, the (p)ppGpp level is tightly regulated by two enzymes, the (p)ppGpp synthetase RelA and the bifunctional synthetase/hydrolase SpoT. To assess the role of CRP in the stringent response, we monitored the (p)ppGpp level in the WT, Δ*crp*, and C*crp* strains during glucose starvation, a condition known to trigger (p)ppGpp accumulation (1). Consistent with the RT-PCR data, we observed that the (p)ppGpp level was dramatically reduced in the Δ*crp* strain (Fig. 1G). Taken together, our results showed that cAMP-CRP triggers (p)ppGpp accumulation in *E. coli*.

### CRP indirectly regulates translation through YfiQ-dependent S1 acetylation

**Fig 2:**
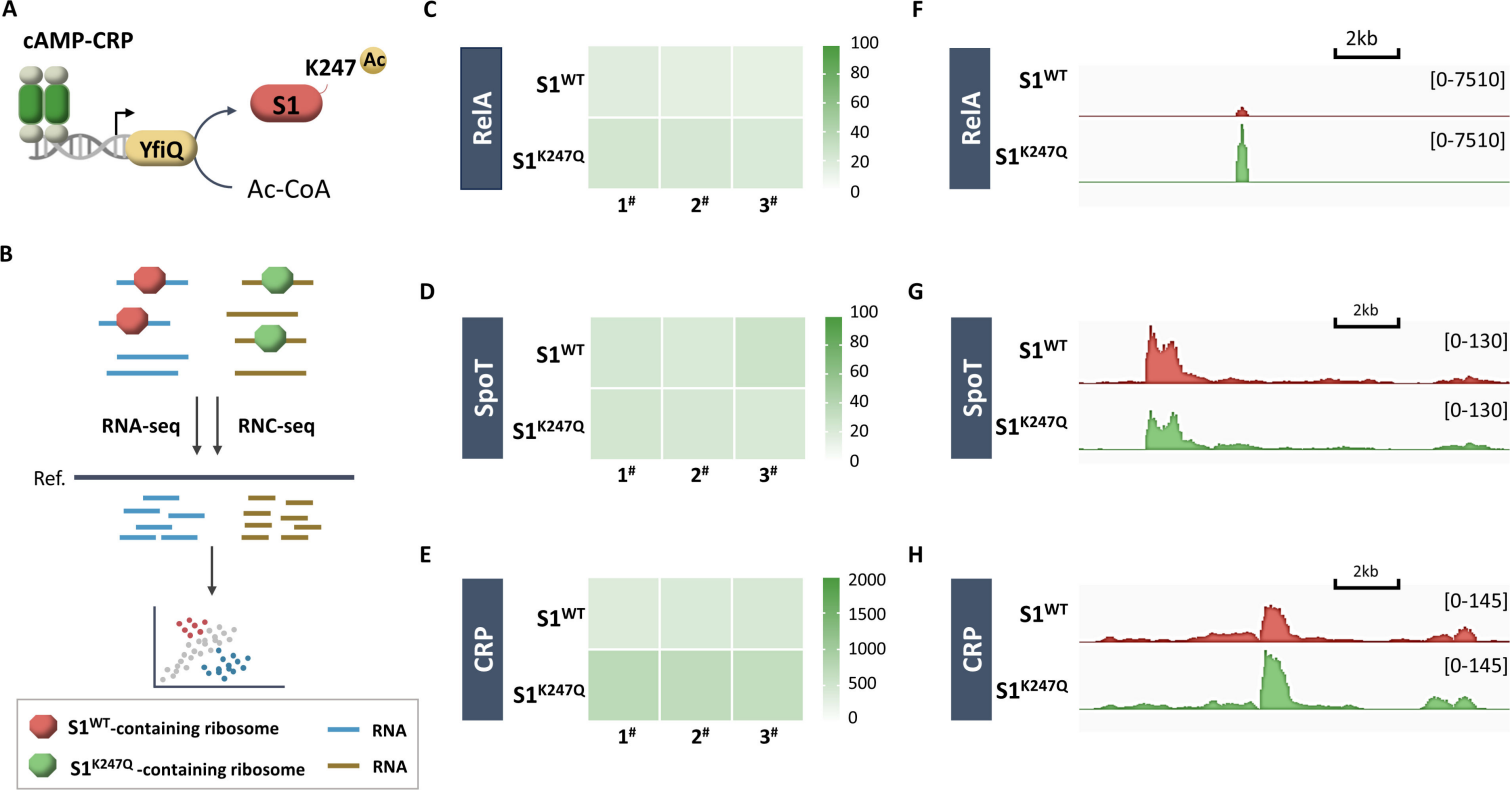
S1 acetylated at K247 preferentially recruits mRNAs of the *relA*, *spoT*, and *crp* genes. (A) cAMP-CRP acts through the acetyltransferase YfiQ to control S1 acetylation. (B) Workflow for RNA-Seq and RNC-Seq of acetyl-mimetic S1 (K247Q) or S1^WT^ containing *E. coli*. Normalized distribution of RNA-Seq reads for the S1^K247Q^ and S1^WT^ strains along the *relA* (C), *spoT* (D), and *crp* (E) genes. Normalized distribution of RNC-Seq reads in the S1^K247Q^ (green) and S1^WT^ (red) strains along the *relA* (F), *spoT* (G), and *crp* (H) genes. *n*  =  3 independent experiments (same donor).

As shown in Fig. 1G, *crp* deficiency almost completely prevented the synthesis of (p)ppGpp during glucose starvation, and CRP-dependent transcriptional regulation did not cause the same degree of repression in *relA* and *spoT* transcription in the Δ*crp* strain (Fig. 1E and F). This result supports the notion that CRP might regulate protein expression at the translational level. One critical step in translation regulation is initiation, which involves the attachment of mRNAs to ribosomes through protein S1 (26). Chemical modification of ribosomes plays an important regulatory role in cellular translation adaptation in response to environmental stresses. Importantly, our previous work (27) revealed that protein S1 acetylation can be catalyzed by the lysine acetyltransferase YfiQ using Ac-CoA as the acetyl donor or nonenzymatically by acetyl phosphate (AcP). AcP induces acetylation of protein S1 during nitrogen limitation, which in turn impacts its binding with distinct mRNAs (27). However, the function of YfiQ-dependent acetylation of the S1 protein has not been determined. Interestingly, YfiQ was previously identified as a target of cAMP-CRP in *E. coli* and is positively regulated by cAMP-CRP (28). It is possible that translational reprogramming manifests as cooperative cAMP-CRP regulatory linkages that activate YfiQ-dependent acetylation of S1 (Fig. 2A). This possibility motivated us to test whether there are measurable translation preferences associated with YfiQ-dependent acetylation of S1 in *E. coli*. Because K247 is the only acetylated site identified in S1 acetylation catalyzed by YfiQ (27), we constructed a prototype with an acetyl-mimetic (K247Q) mutant S1. The corresponding strains S1^K247Q^ and S1^WT^ were selected and subjected to deep transcriptome sequencing (RNA-Seq) and ribosome nascent chain sequencing (RNC-Seq) (Fig. 2B) as described previously (27).

We visualized the reads of the *relA*, *spoT*, and *crp* genes in our sequencing data. The effect of S1 acetylation at K247 determined through RNA-Seq indicated that the mRNA levels of the *relA*, *spoT,* and *crp* genes were similar in S1^K247Q^ and S1^WT^ (Fig. 2C through E). In contrast, the Integrative Genomics Viewer (IGV) plots revealed that the read numbers of the *relA* and *crp* genes were significantly greater in S1^K247Q^ than in S1^WT^, whereas the *spoT* gene exhibited a very small decrease in S1^K247Q^ compared to S1^WT^ (Fig. 2F through H). These results implied that mimicking S1 acetylation at K247 specifically induces basal translation of the *relA* and *crp* genes; similarly, YfiQ-catalyzed S1 acetylation could be predicted to affect RelA and CRP translation, which is directly regulated by cAMP-CRP. Taken together, these observations show that CRP could function as a translation regulator through YfiQ-dependent S1 acetylation. In addition, this translational control could confer a self-activating effect on CRP.

### S1 acetylation at K247 leads to preferential translation of RelA, SpoT, and CRP

**Fig 3:**
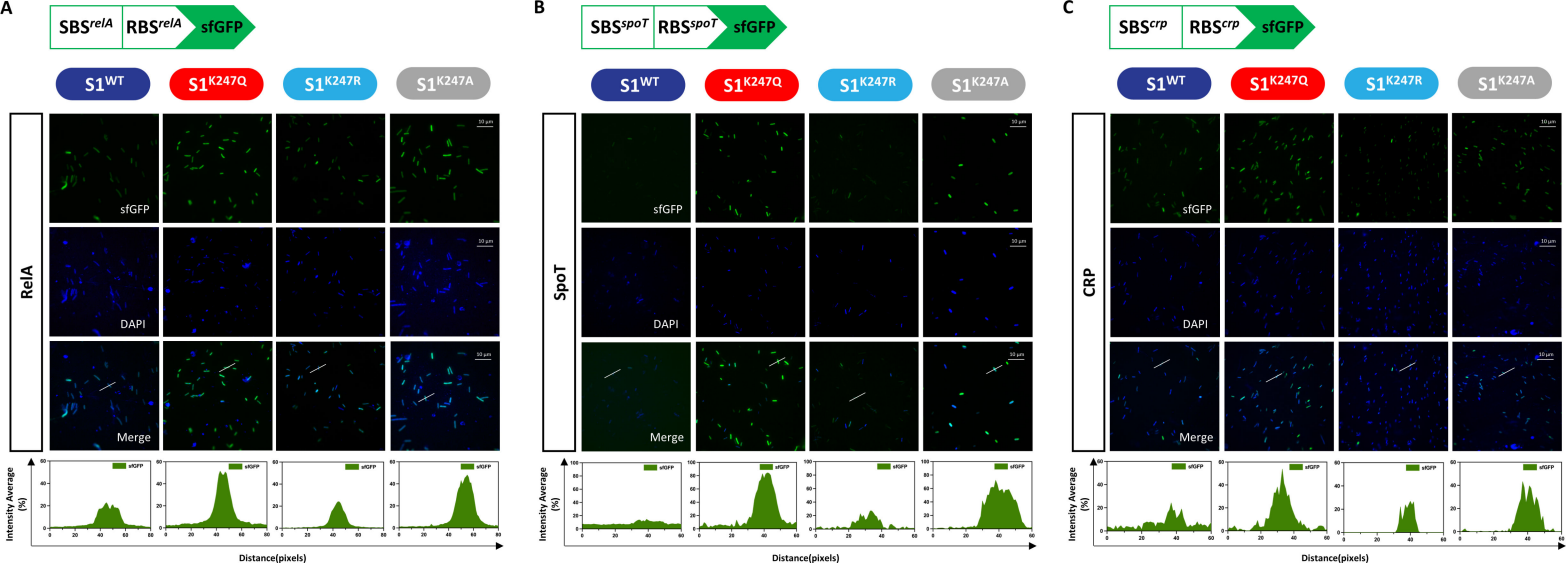
S1 acetylation at K247 leads to preferential translation of (A) RelA, (B) SpoT, and (C) CRP. Fluorescence microscopy of the *E. coli* S1^WT^, S1^K247A^, S1^K247Q^, and S1^K247R^ strains that were engineered to express sfGFP. The strains were grown for 8 h in liquid LB medium at 37°C. Microscopy was replicated twice with similar results. Plots of the arbitrary units along the line indicate the fluorescence intensity in the high-magnification images analyzed using ImageJ. Scale bars, 10 µm.

To investigate whether these translational analysis results were specific to acetylation at K247, we constructed circuits featuring the S1^K247A^, S1^K247Q^, and S1^K247R^ variants based on the principle that alanine (A) simulates inactivation, glutamine (Q) can serve as a structural mimic of acetyl-lysine, and that arginine (R) serves as the genetic mimic of unacetylated lysine (29). Because both the S1-binding sequence (SBS) and ribosome-binding site (RBS) are crucial for S1-mediated translational regulation, we constructed *E. coli* strains overexpressing S1^WT^, S1^K247A^, S1^K247Q^, and S1^K247R^ in which the SBS and RBS of RelA, SpoT, and CRP drove the translation of an sfGFP fluorescent reporter. These translational reporters are transcribed by the same promoter. sfGFP fluorescent protein expression was used as a proxy to quantify the translation of RelA, SpoT, and CRP after exposure to different states of S1. For these twelve strains, we observed a consistent pattern of activation of RelA (Fig. 3A), SpoT (Fig. 3B), and CRP (Fig. 3C) translation during exposure to S1^K247Q^ and S1^K247A^. Quantitative comparisons between the strains also revealed significant increases in the fluorescence intensities of S1^K247Q^ and S1^K247A^ relative to those of S1^WT^ and S1^K247R^ (Fig. 3). Altogether, we reasoned that S1 acetylation at K247 is the likely source of translational induction of RelA, SpoT, and CRP, an effect that is regulated by CRP.

### S1 acetylation at K247 induces (p)ppGpp levels

**Fig 4:**
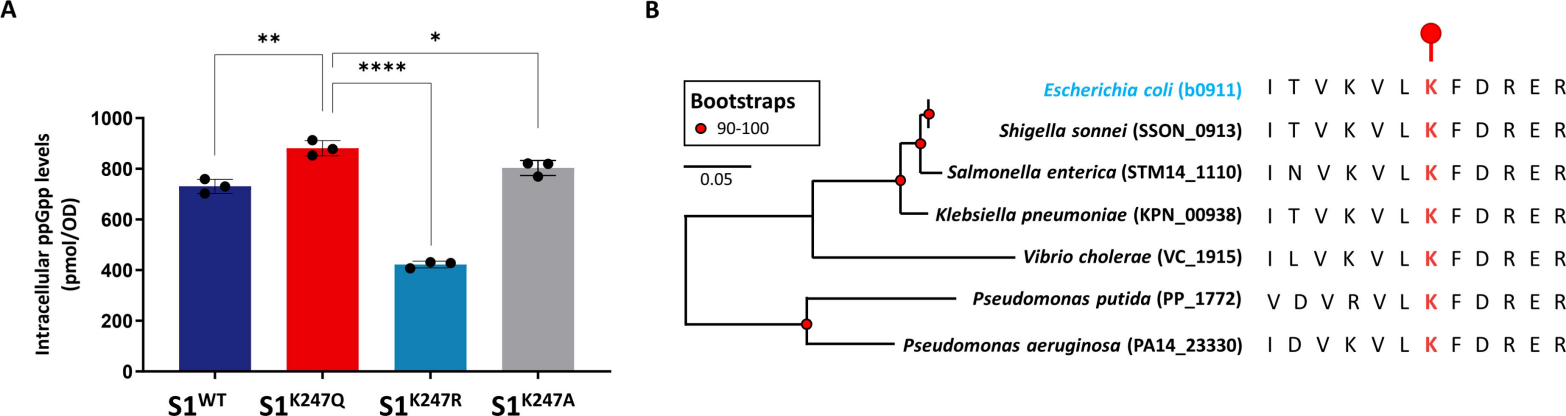
K247 acetylation induces (p)ppGpp levels. (A) Intracellular (p)ppGpp concentrations in cell extracts of the *E. coli* S1^WT^, S1^K247A^, S1^K247Q^, and S1^K247R^ strains grown in liquid LB medium during late exponential growth. The (p)ppGpp concentrations of the samples were normalized to the OD_600_. The error bars show the standard deviations (SDs) of three independent experiments, and the mean values from independent experiments were used. An ordinary one-way analysis of variance (ANOVA) was performed to test the significance. *P < 0.1, **P < 0.01, ****P < 0.0001. (B) Sequence alignment of S1 proteins within *E. coli*, *S. enterica*, *K. pneumoniae*, *S. sonnei*, *V. cholerae*, *P. putida*, and *P. aeruginosa*. The conserved K247 site is in red.

To gain insight into whether such mutations affect intracellular (p)ppGpp levels, we tested cellular (p)ppGpp concentrations in the *E. coli* S1^WT^, S1^K247A^, S1^K247Q^, and S1^K247R^ strains. Consistent with our observed experimental results for fluorescence intensity, the *E. coli* S1^K247Q^ and S1^K247A^ strains exhibited an increase in the intracellular level of (p)ppGpp compared with that of S1^WT^, whereas the S1^K247R^ strain showed a significant reduction in the (p)ppGpp concentration (Fig. 4A). We concluded that K247 present in S1 is important for maintaining the intracellular (p)ppGpp level and that acetylation at K247 strongly contributes to (p)ppGpp homeostasis. Furthermore, a phylogenetic survey was created to complement the possibility that K247 acetylation might broadly exist. S1 homologs from *Salmonella enterica* (STM14_1110), *Klebsiella pneumoniae* (KPN_00938), *Shigella sonnei* (SSON_0913), *Vibrio cholerae* (VC_1915), *Pseudomonas putida* (PP_1772), and *Pseudomonas aeruginosa* (PA14_23330) were screened as representative bacteria. Indeed, according to the alignment of the *E. coli* S1 sequence with its homologs, K247 was highly conserved (Fig. 4B). We predict, therefore, that the conserved residue K247 is important for the S1 function involved in translational regulation in other bacteria.

### Regulation of (p)ppGpp homeostasis by CRP is dependent on glucose availability

**Fig 5:**
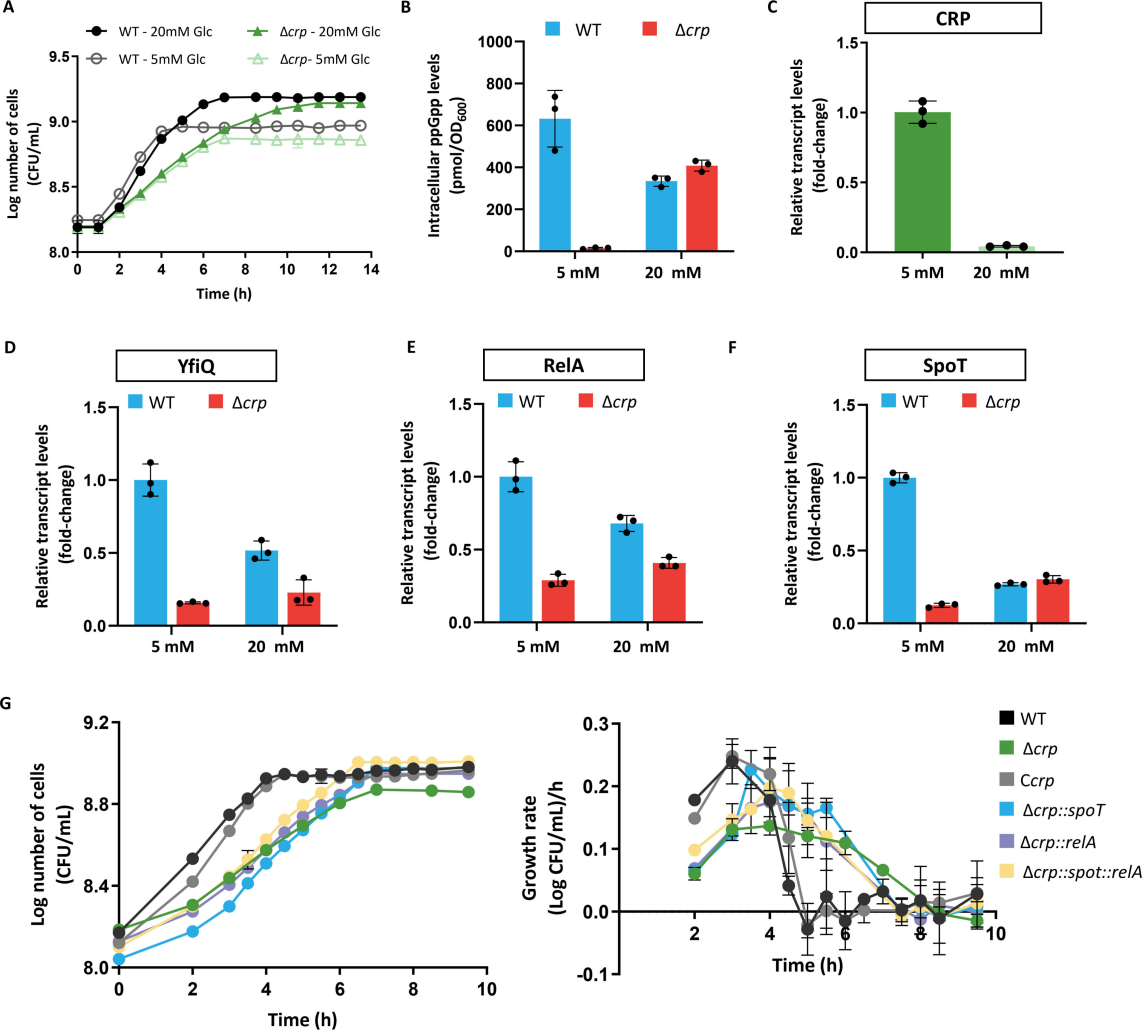
CRP regulates the basal level of (p)ppGpp during glucose downshift. (A) Growth curves of *E. coli* strains cultured in standard glucose or limited glucose. (B) Intracellular (p)ppGpp concentrations in cell extracts of *E. coli* strains cultured in standard glucose or limited glucose until late exponential growth. The transcription levels of *crp* (C), *yfiQ* (D), *relA* (E), and *spoT* (F) in *E. coli* strains grown until late exponential growth in standard glucose or limited glucose. The fold change represents the expression level compared to that in the WT strain cultured in limited glucose. The error bars show the standard deviations (SDs) of three independent experiments, and mean values from independent experiments were used. The mean value in the WT strain cultured in 5 mM glucose was set to 1.0 (arbitrary units). (G) Growth curves and growth rates of *E. coli* strains cultured in limited glucose.

Microorganisms constantly face challenges posed by fluctuating conditions in natural environments, and glucose is generally utilized preferentially via CCR (30). Once glucose is depleted, bacterial growth is transiently arrested, and the cellular concentrations of (p)ppGpp display an abrupt increase (31). The accumulated (p)ppGpp thus controls regulatory networks that coordinate the resumption of growth (32). Considering that the CCR is determined by cAMP-CRP, it is reasonable to assume that (p)ppGpp homeostasis regulated by cAMP-CRP requires stimulation of glucose availability. To verify this, we examined the effect of CRP on the (p)ppGpp level in *E. coli* during growth under glucose starvation conditions (with 5 mM glucose as carbon resource). Both the WT and Δ*crp* strains exhibited growth inhibition during glucose limitation (Fig. 5A). As shown in Fig. 5A, the Δ*crp* mutant had a longer exponential growth phase and exhibited less efficient growth than the WT strain. The (p)ppGpp levels of the *E. coli* WT and Δ*crp* strains during growth in standard glucose (20 mM) or limited glucose (5 mM) conditions indicated that the CRP-induced increase in (p)ppGpp occurred only during glucose starvation (Fig. 5B). This situation induced elevated levels of CRP (Fig. 5C), which indicated that CRP is required to maintain (p)ppGpp levels during glucose starvation. When grown in 5 mM glucose, the Δ*crp* mutant consistently showed significantly reduced transcription of *yfiQ* (Fig. 5D), *relA* (Fig. 5E), and *spoT* (Fig. 5F), which underscores the role of glucose limitation as a stimulatory signal for the CRP-mediated activation of (p)ppGpp accumulation. Loss of CRP causes large shifts in the transcriptomes of key metabolic pathways, and (p)ppGpp controls the bacterial growth rate by reprogramming transcription. To determine whether the decrease in (p)ppGpp is involved in the growth defect of the Δ*crp* mutant, we monitored the growth of the Δ*crp* mutant overexpressing *relA* or *spoT*. Indeed, we observed that the growth of the Δ*crp* mutant was partially restored by the overexpression of *relA* (Fig. 5G, purple line) or *spoT* (Fig. 5G, blue line), indicating that the phenotype of the Δ*crp* mutant is correlated with a low (p)ppGpp level, to a certain extent. These results suggest that CRP functions as a direct link between glucose availability and the RelA/SpoT-mediated stringent response. Finally, we observed that the induction of *relA* and *spoT* could partially complement the auxotrophic phenotype of the Δ*crp* strain (Fig. 5G, yellow line). Taken together, these data suggest that CRP regulates the basal level of (p)ppGpp during glucose downshift.

### (p)ppGpp mediates a negative feedback circuit for CRP activation

**Fig 6:**
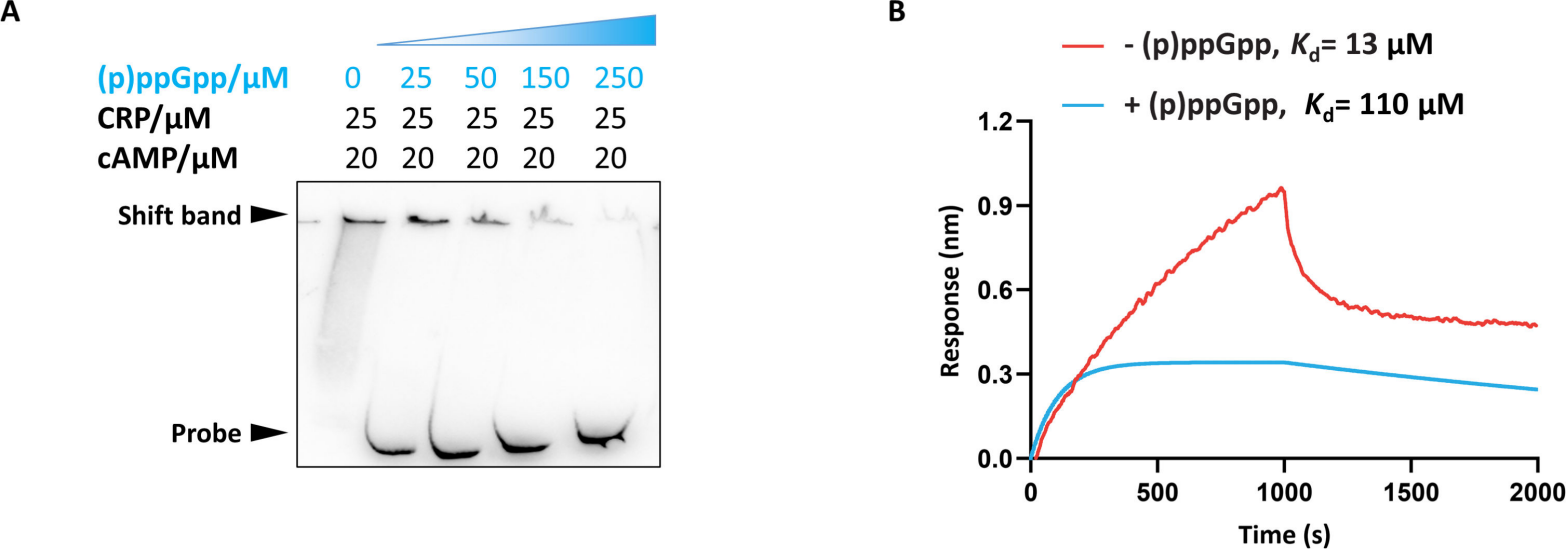
(p)ppGpp inhibits the cAMP-dependent DNA-binding activity of CRP. (A) cAMP-dependent DNA binding with CRP in the presence of increasing amounts of (p)ppGpp. (B) Biolayer interferometry assay of cAMP-CRP with the *relA* gene promoter in the presence (blue) and absence (red) of (p)ppGpp.

The (p)ppGpp–CRP interaction has been reported *in vitro* with a dissociation constant *K*_d_ of 35 µM (33), and the *K*_d_ value is well within the physiological (p)ppGpp concentration range given that the cellular level of (p)ppGpp corresponds to 1 mM during the stringent response (34). cAMP binds to CRP with a dissociation constant *K*_d_ of approximately 20 μΜ (35, 36). Hence, we sought to delineate the function by which (p)ppGpp binds to CRP by monitoring the effects of increasing amounts of (p)ppGpp on the DNA-binding ability of cAMP-CRP (Fig. 6A). There was a significantly weak DNA binding in a dose-dependent manner in the presence of (p)ppGpp (Fig. 6A), indicating that (p)ppGpp dominated the impairment of CRP DNA-binding activity. Next, we determined the affinity of cAMP-CRP for DNA before and after (p)ppGpp addition. Biolayer interferometry (BLI) revealed the very weak affinity of cAMP-CRP for DNA in the presence of (p)ppGpp, which led to a ∼10-fold decrease in DNA-binding affinity (*K*_d_ value from 13 to 110 µM). These findings suggest that (p)ppGpp and cAMP compete for binding to CRP and that high concentrations of (p)ppGpp provide competitive advantages in the CRP interaction. The amino acids of CRP involved in the interaction with cAMP are Gly71, Glu72, Arg82, Ser83, Thr127, and Ser128 of CRP (37); therefore, we hypothesized that these residues might participate in the competition between (p)ppGpp and cAMP for binding to CRP. Overall, these data proposed that (p)ppGpp decreased the affinity of CRP for DNA through competition with cAMP, thus leading to negative feedback and autoinhibition to avoid continuous accumulation of (p)ppGpp *in vivo*.

### A CRP-mediated dual enhancement (CMDE) element facilitates stable activation in synthetic circuits

**Fig 7:**
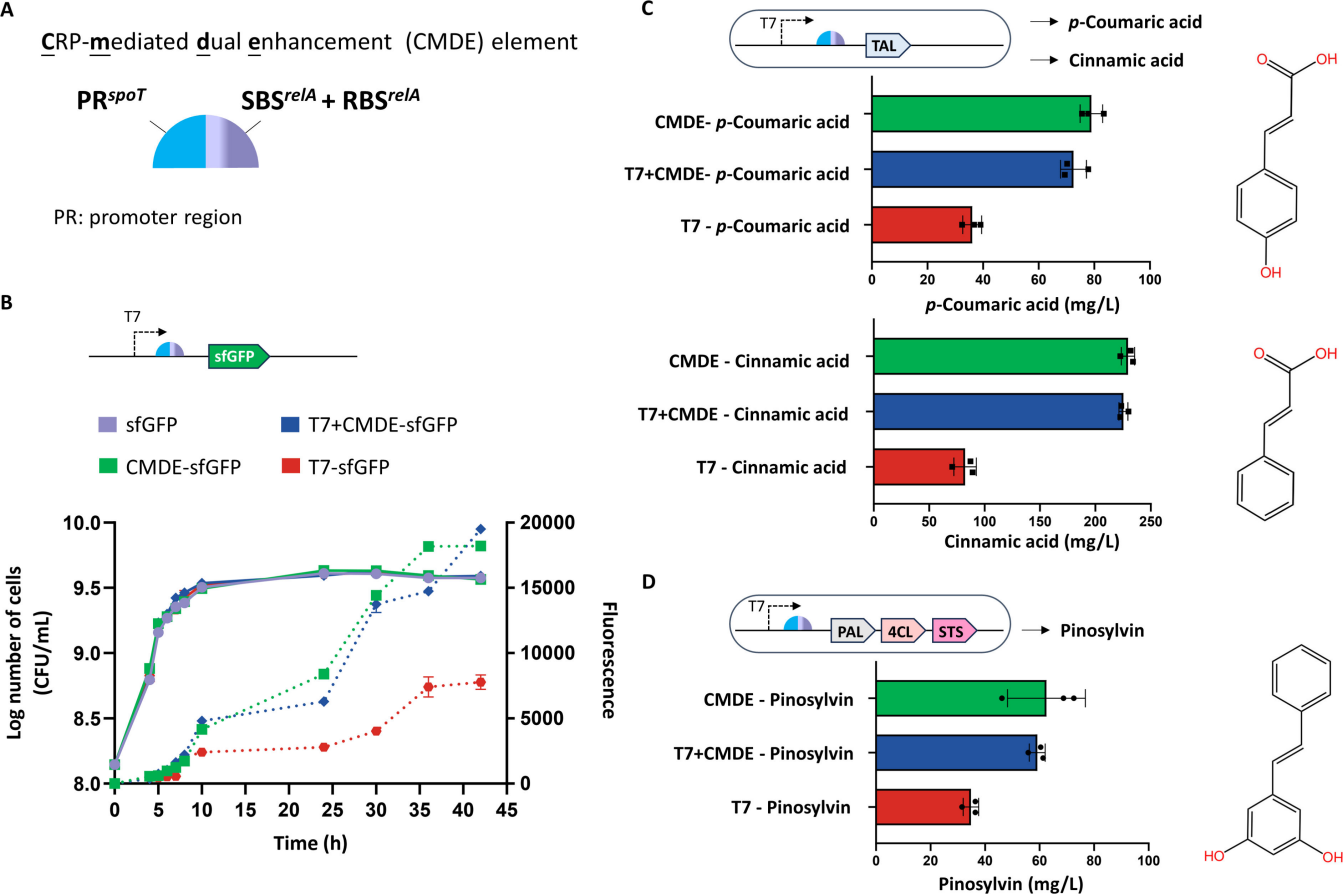
Using the CMDE assembly to engineer synthetic circuits. (A) Schematic of the CMDE element. CMDE is composed of the promoter region of *spoT* and the SBS and RBS regions of *relA*. (B) Growth behavior and quantitative determination of sfGFP reporter expression in the indicated strains. *E. coli* strains were engineered with a no-promoter circuit, a T7-only circuit, a CMDE-only circuit, or a T7-coupled CMDE circuit, and circuit activation was quantified by measuring sfGFP reporter fluorescence. The strains were grown in liquid LB medium at 37°C. (C) *p*-Coumaric acid/cinnamic acid production of engineered *E. coli* strains grown in liquid LB medium at 37°C. (D) Pinosylvin production by engineered *E. coli* strains grown in liquid LB medium supplemented with cerulenin at 37°C. The error bars show the standard deviations (SDs) of three independent experiments.

Our results so far provide strong evidence that CRP acts as a ‘‘trigger’’ to drive the expression of *relA* and *spoT* through two modes: classical transcriptional activation, and YfiQ-dependent acetylation of S1, which autoregulates its own translation of CRP and activates the translation of RelA and SpoT. The presence of both circuits in this model is strongly correlated with glucose depletion, and we hypothesized that using cooperative two-mode assemblies would enable genetic stability in the synthesis of target products during glucose downshift. To test this hypothesis, we created a simple CMDE element designed to simulate the expression of target genes harboring both translational and transcriptional circuits of CRP. Because CRP strongly affects *spoT* transcription and *relA* translation, this element is composed of the promoter region of *spoT* and the SBS and RBS regions of *relA* (Fig. 7A). The T7 promoter was used as a control, and circuit activation was quantified by sfGFP reporter fluorescence. Following circuit induction, we observed a dramatic fluorescence enhancement in the CMDE-containing circuit relative to the T7-only control, and the CMDE-only circuit maintained a reporter activation that was similar to that of the coupled circuit (with the integrated T7 promoter and the CMDE cassette) (Fig. 7B).

We next sought to use CMDE to engineer the circuit architecture of *p*-coumaric acid/cinnamic acid to determine whether it could provide the same promotion effect in a synthetic circuit. As shown in Fig. 7C, the same patterns of synthetic activation were observed with circuits featuring the CMDE or T7 promoter coupled with the CMDE. The CMDE-only circuit maintained the production of *p*-coumaric acid/cinnamic acid in a manner similar to that of the coupled circuit, which exhibited a remarkable more than twofold greater yield of the products relative to that of the T7-only control, suggesting the maintenance of genetic stability and robust activation. Of note, CMDE application resulted in a cinnamic acid titer of 230 ± 7 mg/L, which is a very high titer in engineered *E. coli* under cultivation conditions. We expanded the existing CMDE cassette design to more complex circuit architectures. In artificial *E. coli* engineered for pinosylvin synthesis (38), the role of CMDE in maintaining circuit activity was further demonstrated by a twofold increase in pinosylvin production in CMDE-containing circuits (Fig. 7D). Taken together, these results reinforce functional CMDE as an attractive strategy for synthetic circuit stability and, furthermore, indicate that this strategy has the potential to address biotechnological needs.

## DISCUSSION

Dynamic modulation of the (p)ppGpp alarmone level is paramount for maintaining cellular homeostasis. Here, we showed that cAMP-CRP, which possesses active RelA and SpoT, relies on cellular glucose availability, revealing that in addition to its well-known function as a transcription regulator, CRP has a noncanonical role in translation regulation. These dual mechanisms enable a rapid response to glucose limitation and fast synchronization between the entire cellular population of the enzymes RelA and SpoT. A feedback response in which (p)ppGpp negatively controls the activity of CRP occurs. As noted above, the cAMP–CRP interaction is inhibited by (p)ppGpp, which accounts for the reduced cAMP-CRP binding associated with high levels of (p)ppGpp (Fig. 6). Once excess (p)ppGpp is produced to trigger a negative feedback response, the cell commits to stringency, rapidly traversing intermediate (p)ppGpp levels and thus ensuring a fast coordinated response at the cellular level, which is similar to bistable switches.

**Fig 8:**
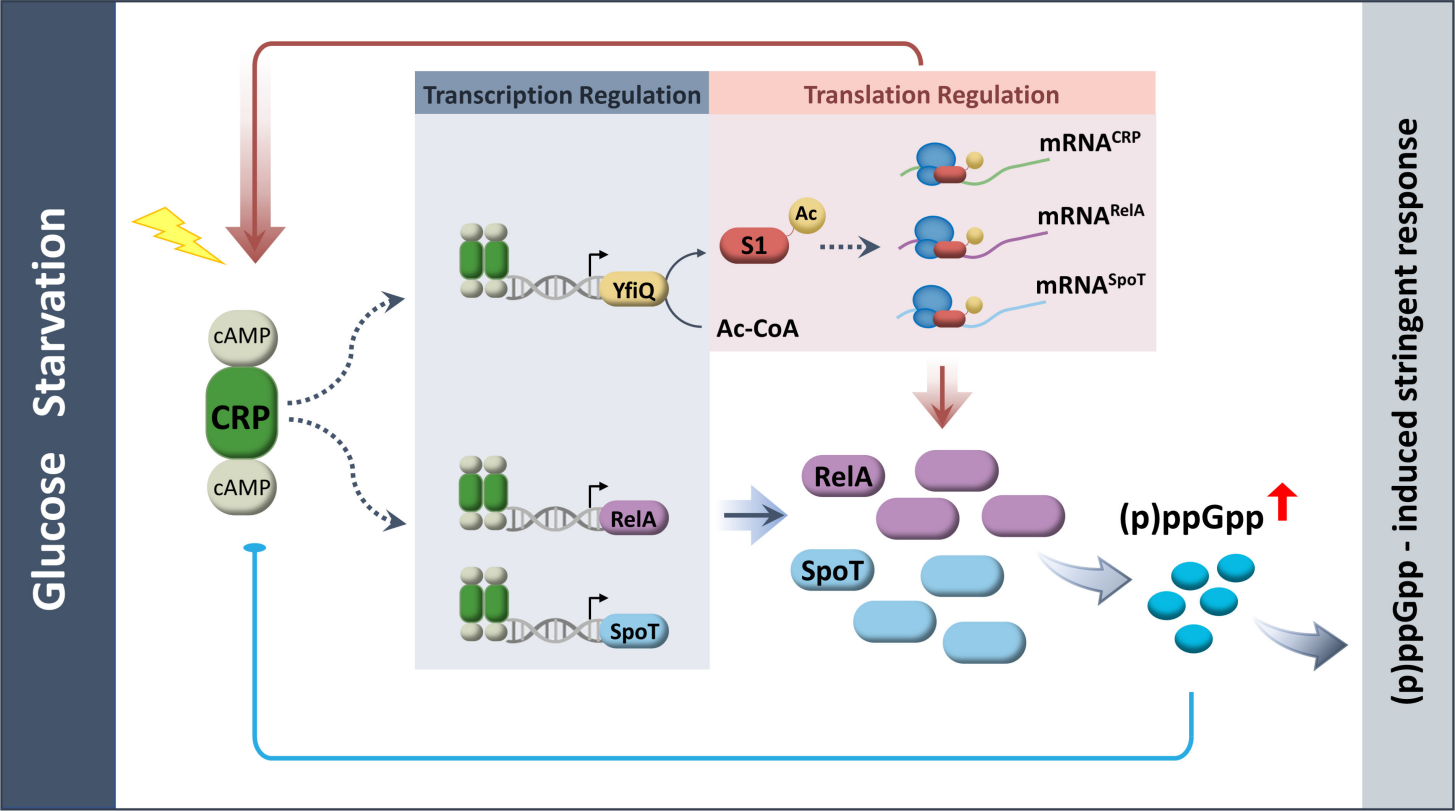
Model of a functional dual program relying on CRP for proper (p)ppGpp homeostasis.

Positive regulation at the translational level is inherently faster than positive regulation at the transcriptional level, as *de novo* production of mRNA is not needed. The activation of RelA/SpoT and CRP translation entails S1 acetylation at K247, which is catalyzed by YfiQ. These results support the view that YfiQ-dependent acetylation also triggered by CRP is coupled to glucose-limiting conditions, allowing more precise control over translation, which extends our previous work (27). Although it is difficult to directly compare the relative importance of the role of CRP in translation regulation to its role in transcription regulation, our results show that K247 of S1 is conserved and worthy of further study. In addition, the cAMP-CRP complex indirectly regulates SpoT-dependent accumulation of (p)ppGpp and cell survival through YtfK (22, 39). The effects of CRP span a continuum from survival responses elicited by stress, such as glucose deprivation, to the housekeeping role of basal (p)ppGpp levels in *E. coli*. In terms of the similar roles of CRP and (p)ppGpp in genetic reprogramming in response to stressful conditions, our results offer a framework that CRP-mediated activation is complementary to the effects of the (p)ppGpp-dependent stringent response (Fig. 8).

The (p)ppGpp-mediated stringent response is well documented for accomplishing optimal resource allocation upon facing environmental shifts, and the stringent response is also linked to the production of secondary metabolites, including antibiotics, in *Streptomycetes* (40). Over the last two decades, the engineering of artificial transcriptional regulatory networks to reprogram cellular behavior has become a major focus in the field of synthetic biology and has emerged as a powerful approach for the development of cell-based biotechnologies (41–45). These engineered networks are constructed using transcriptional regulator interactions that specify links between gene expression outputs and molecular inputs such as proteins, RNA, or small molecules (46–49). We thus investigated and confirmed the design of a CRP-mediated self-activating feedback loop, which is central to the function of numerous natural and engineered networks. From a synthetic biology perspective, our work demonstrates that CMDE assembly is a robust, generalizable strategy for engineering synthetic circuits that enable stable activation. Moreover, CMDE has been proven to be small, simple, and flexible, with a fragment length of less than 600 bp. The self-activating feedback feature also eliminates additional costs from adding an inducer molecule. This capability would be valuable in biomanufacturing applications, where protein or chemical production can be improved by inducing discrete expression of biosynthetic pathway genes. Finally, in addition to identifying a surprising role for CRP in (p)ppGpp homeostasis, we demonstrated that the design strategies we developed here could be translated more broadly to other molecular settings.

## MATERIALS AND METHODS

### Strains and growth conditions

All of the strains used in this study are listed in Table S1 and were cultured in LB (Luria-Bertani) medium or M9 medium (6.8 g of Na_2_HPO_4_, 3.0 g of KH_2_PO_4_, 0.5 g of NaCl, 1.0 g of NH_4_Cl, and 5 or 20 mM glucose in 1 L ddH_2_O) at 37°C with shaking at 220 rpm. The corresponding culture conditions used were based on the experimental needs.

### Creation and identification of mutants

The Δ*crp* strain was generated by replacing the *crp* gene locus with a kanamycin resistance (*kanR*) cassette through homologous recombination via the λ-Red system as previously described (50). The *kanR* fragments were PCR amplified from the pET28a plasmid with the primers *crp*-ko-F1/R1 and *crp-kanR*-F/R (Table S1), which contain a 59 bp homologous extension upstream and downstream of the *crp* coding sequence. The *kanR* fragments were subsequently introduced into *E. coli* MG1655::pKD46 electrocompetent cells with an electric field intensity of 3 kV (15 kV/cm) for 4 ms. The cells were subsequently resuspended in 800 µL of LB medium immediately after the electroporation pulse and were cultured at 37°C for 2–3 h. Diagnostic PCR was conducted to confirm the successful deletion of the *crp* gene.

For complementary strains, *crp*/*relA*/*spoT* fragments were PCR amplified from *E. coli* MG1655 genomic DNA and were inserted into pWT021a through the primers pWT-F/R. These plasmids were subsequently transformed into electrocompetent Δ*crp* cells, which were subsequently spread on LB solid plates supplemented with 50 mg/L kanamycin and 50 mg/L streptomycin.

### Protein expression and purification

The recombinant plasmid pProEX-*crp* was transformed into *E. coli* BL21 (DE3). The *E. coli* was subsequently grown in LB medium supplemented with 100 mg/L ampicillin to an optical density (OD_600_) of 0.6. Protein expression was induced with 0.5 mM isopropyl-β-D-thiogalactopyranoside (IPTG) and then incubated at 18°C for 16 h. Then, the cells were centrifuged at 7,000 × *g* at 4°C for 20 min. The His-tagged protein was purified by Ni-NTA affinity chromatography (Transgene) and eluted with 250 mM imidazole (in 50 mM NaH_2_PO_4_, 300 mM NaCl, pH 8.0). The purified proteins were analyzed by SDS‒PAGE, and the protein concentration was determined with a bicinchoninic acid (BCA) protein assay kit (Transgene).

### Real-time RT-PCR

Total RNA extraction was performed according to our previous work (51). Total RNA (1 µg) was used to synthesize cDNA using the PrimeScript RT Reagent Kit with gDNA Eraser (Takara). 16S rRNA was used as the internal standard. The resulting cDNA was diluted and used as a template for real-time RT-PCR with SYBR Premix Ex Taq GC (Takara). The primers used are listed in Table S2. PCR assays were performed on a CFX96 real-time system (Bio-Rad, Hercules, CA, USA) with the following PCR conditions: 95°C for 5 min; then 40 cycles of 95°C for 5 s and 60–64°C for 30 s; and an extension at 72°C for 10 min. The transcriptional variations were analyzed by the threshold cycle (2^-∆∆CT^) method.

### (p)ppGpp quantification

Bacteria were grown at 37°C overnight in M9 medium supplemented with 5 or 20 mM glucose until late exponential growth. The cells were harvested by centrifugation at 7,000 × *g* for 2 min at 4°C and were washed twice with 1× phosphate-buffered saline (PBS). Then, the bacteria were resuspended in 1 M freezing formic acid, and (p)ppGpp was extracted at 4°C for 1 h. After centrifugation at 8,000 × *g* at 4°C for 20 min, the liquid supernatant was collected. After the supernatant was frozen in liquid nitrogen, it was placed in a vacuum freeze dryer for 2 d. The freeze-dried powder was dissolved in 1 mL of ddH_2_O, and the amount of (p)ppGpp was measured via a Microorganism GT ELISA Kit (Shanghai Enzyme-linked Biotechnology Co., Ltd.). The intracellular (p)ppGpp concentrations were converted to picomoles per OD.

### Electrophoretic mobility shift assay (EMSA)

The DNA fragments, the upstream regions of the detected genes, used for EMSA were amplified by PCR. The primers used for amplification are listed in Table S2 and contain a universal sequence (5′-AGCCAGTGGCGATAAG-3′) for biotin labeling of EMSA probes. Biotin-labeled probes were generated via PCR with a 5’ biotin-modified universal primer and then purified with a PCR purification kit (Shanghai Generay Biotech Co., Ltd., China). Probes were quantified beforehand with a microplate reader (Biotek, Winooski, VT, USA). A biotin-labeled probe was incubated at 25°C for 30 min with a concentration gradient of purified CRP protein in 1× EMSA/Gel-Shift Binding Buffer (Beyotime Biotechnology). The samples were separated on a 6% nondenaturing PAGE gel at 4°C in 0.5× Tris-borate-EDTA and 200 µM cAMP buffer. The signals were detected with a BeyoECL Plus kit (Beyotime Biotechnology).

### Live-cell imaging

Freshly grown cells were picked and resuspended in PBS buffer. The samples were dyed with DAPI (4′,6-diamidino-2-phenylindole) for 10 min at room temperature. The washed and resuspended cells were transferred to microscope slides and dried. Once dry, the microscope slides were covered with coverslips. A Nikon N-SIM S Super-Resolution microscope was used for live-cell imaging. All fluorescence images were obtained using the same equipment settings for comparison purposes. Images were processed with NIS Elements AR software.

### Measurement of cinnamic acid and *p*-coumaric acid concentrations

Cinnamic acid and *p*-coumaric acid standards were purchased from Macklin. The samples and standards were analyzed via a high-performance liquid chromatography (HPLC) system (Shimadzu) equipped with a reverse-phase C18 column (a Diamonsil Plus 5 µm C18-A, 250 × 4.6 mm, DiKMA) and an ultraviolet-visible (UV-VIS) detector. For HPLC analysis, phase A was an ammonium formate solution (20 mM, pH 3), and phase B was acetonitrile (ACN). The initial ACN concentration was 2% (vol/vol), which was linearly increased to 40% (vol/vol) after 10 min, linearly decreased to 20% (vol/vol) after 15 min, subsequently decreased to 2% (vol/vol) after 18 min, and maintained at 2% for 20 min. The column was maintained at 40°C with a flow rate of 1 mL/min throughout the process. Cinnamic acid or *p*-coumaric acid was detected with a 289-nm excitation wavelength. The concentration was calculated by determining the HPLC peak area according to a standard curve. All measurements were performed in triplicate.

### Pinosylvin determination

The extraction and HPLC analysis of pinosylvin were performed as previously described (38). In brief, 100 µL of cell-free supernatant was mixed with the same volume of ice-cold ACN, and the mixture was centrifuged at 8,000 × *g* for 5 min. A total of 80 µL of the mixture was loaded onto an Xbridge C18 column (4.6 × 250 mm, Waters, Milford, MA USA). The two mobile-phase solvents used were buffer A (96.96/0.04, water/formic acid, vol/vol) and buffer B (ACN). The initial gradient was 98% solvent A and 2% solvent B at 0.7 mL/min. At 4 min, solvent B was gradually increased to 50% over 6 min. The UV detector was set at 308 nm for pinosylvin.

### Biolayer interferometry (BLI) assay

A streptavidin (SA) biosensor purchased from ForteBio was used (51) in this work. The loading buffer (pH 8.0) contained 10 mM HEPES, 2 mM MgCl_2_, 0.1 mM EDTA, and 200 mM KCl, and the running buffer contained an extra 10 µg/mL BSA and 0.02% Tween-20. The biotin-labeled DNA probe used was the same as that used for the EMSAs. The DNA probe was stored in loading buffer, and His-tagged protein was stored in running buffer during the BLI assay with SA sensors. The samples were then detected within the OptiPlate-96 Black Opaque (PerkinElmer).

### RNA-seq and RNC-seq

RNA-seq and RNC-seq were performed with three biological replicates. The *E. coli* strains were grown in 100 mL of LB at 37°C and 220 rpm until late exponential growth. The cells were harvested immediately by centrifugation at 4°C, quickly frozen on dry ice and subsequently sent to Chi Biotech Co., Ltd., for further treatment. The samples were treated with prechilled cell lysis buffer (20 mM HEPES-KOH, pH 7.4), 1% Triton X-100 in ribosome buffer (RB buffer), 200 mM KCl, 15 mM MgCl_2_, 100 µg/mL cycloheximide, and 2 mM dithiothreitol. The cell lysates were harvested and transferred to prechilled 1.5 mL tubes after incubation on ice for 30 min. Then, the cell debris was removed by centrifugation at 4°C for 20 min. The supernatants were transferred to the surface of sucrose buffer (30% sucrose in RB buffer) and then ultracentrifuged at 4°C by a T-865 fixed angle rotor (Thermo Fisher) at 42,500 rpm for 5 h. The pellets were then resuspended in RB buffer. RNC-RNA and total RNA were isolated with TRIzol Reagent (Invitrogen, USA) according to the manufacturer’s instructions. The sequencing libraries were generated via an NEBNext Ultra RNA Library Prep Kit for Illumina (NEB, USA, Catalog #: E7530L) following the manufacturer’s instructions and were sequenced using an Illumina NovaSeq 6000.

### RNA-seq analysis

The quality control of the RNA-Seq data was conducted using FastQC with default parameters. Clean paired-end reads were aligned to the *E. coli* K-12 MG1655 reference genome sequence via STAR (2.5.3a). The count for each gene was calculated using featureCounts (52), and then normalized with the FPKM (fragments per kilobase million) method. To identify differentially expressed genes, edgeR (53) was used, and a false discovery rate (FDR) < 0.01 was set as the threshold. Finally, visualization of RNC-Seq tracks was performed using IGV tracks (54).

### RNC-seq analysis

The raw data of six samples were demultiplexed and analyzed further. First, all reads were processed with Cutadapt to remove adapter sequences and low-quality reads, and then aligned to the reference genome (*E. coli* K-12 MG1655) by STAR (2.5.3a) with default parameters. Second, the count for each gene was calculated using featureCounts and normalized via the FPKM method. Differentially expressed genes with an FDR < 0.05 were identified via DESeq2 (55). Finally, visualization of the RNC-Seq tracks was performed using IGV tracks.

## Data Availability

RNA-seq and RNC-seq raw data are available in the GEO database under accession ID GSE254165 and GSE254167. All study data are included in the article and/or supplemental material.

## SUPPLEMENTAL MATERIAL

Supplemental material mbio.02430-24-s0001.docx Supplemental figures and tables.

ASM does not own the copyrights to Supplemental Material that may be linked to, or accessed through, an article. The authors have granted ASM a non-exclusive, world-wide license to publish the Supplemental Material files. Please contact the corresponding author directly for reuse.
