# Supplementary material for: A dual program for CRP-mediated regulation in bacterial alarmone (p)ppGpp
Source: mBio. 2024 Oct 4;15(11):e02430-24. doi: 10.1128/mbio.02430-24 (PMC11559003; doi:10.1128/mbio.02430-24)
Supplement: Supplemental material — Supplemental figures and tables. [file mbio.02430-24-s0001.docx]

**A dual program for CRP-mediated regulation in bacterial alarmone (p)ppGpp**

Li Zhao^1^, Shi-Yu Zhou^1^, Yu Fu^1^, Jin-Long Shen^1^, Bin-Cheng Yin^1^, Di You^1^*, Bang-Ce Ye^1,2^*

^1^Lab of Biosystems and Microanalysis, State Key Laboratory of Bioreactor Engineering, East China University of Science and Technology, Shanghai 200237, China

^2^Institute of Engineering Biology and Health, Collaborative Innovation Center of Yangtze River Delta Region Green Pharmaceuticals, College of Pharmaceutical Sciences, Zhejiang University of Technology, Hangzhou 310014, Zhejiang, China

* Corresponding authors

**Corresponding authors**

Di You

Associate Professor, Lab of Biosystems and Microanalysis,

State Key Laboratory of Bioreactor Engineering,

East China University of Science and Technology, Shanghai 200237, China

Tel/Fax: 0086-21-64253832

Email: 030111115@mail.ecust.edu.cn

Bang-Ce Ye

Professor, Cheung Kong Scholar the Ministry of Education,

State Key Laboratory of Bioreactor Engineering,

East China University of Science and Technology, Shanghai 200237, China

Tel/Fax: 0086-21-64252094

Email: [bcye@ecust.edu.cn](mailto:bcye@ecust.edu.cn)


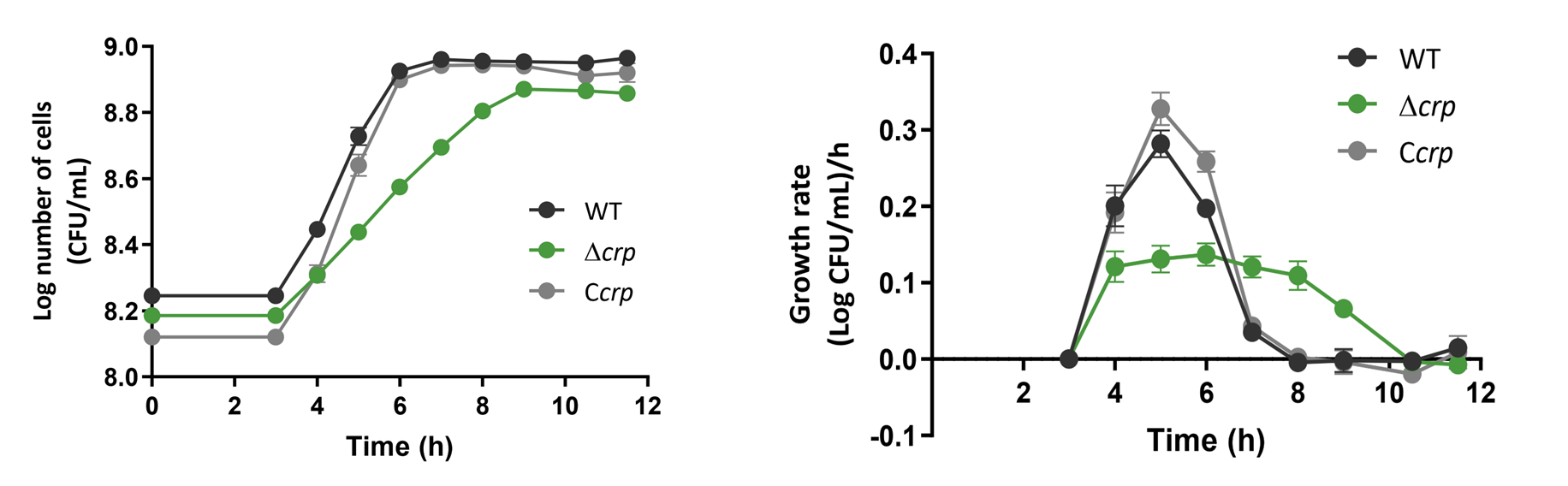


**Figure S1**. Growth curves and growth rates of *E. coli* strains cultured in limited glucose.


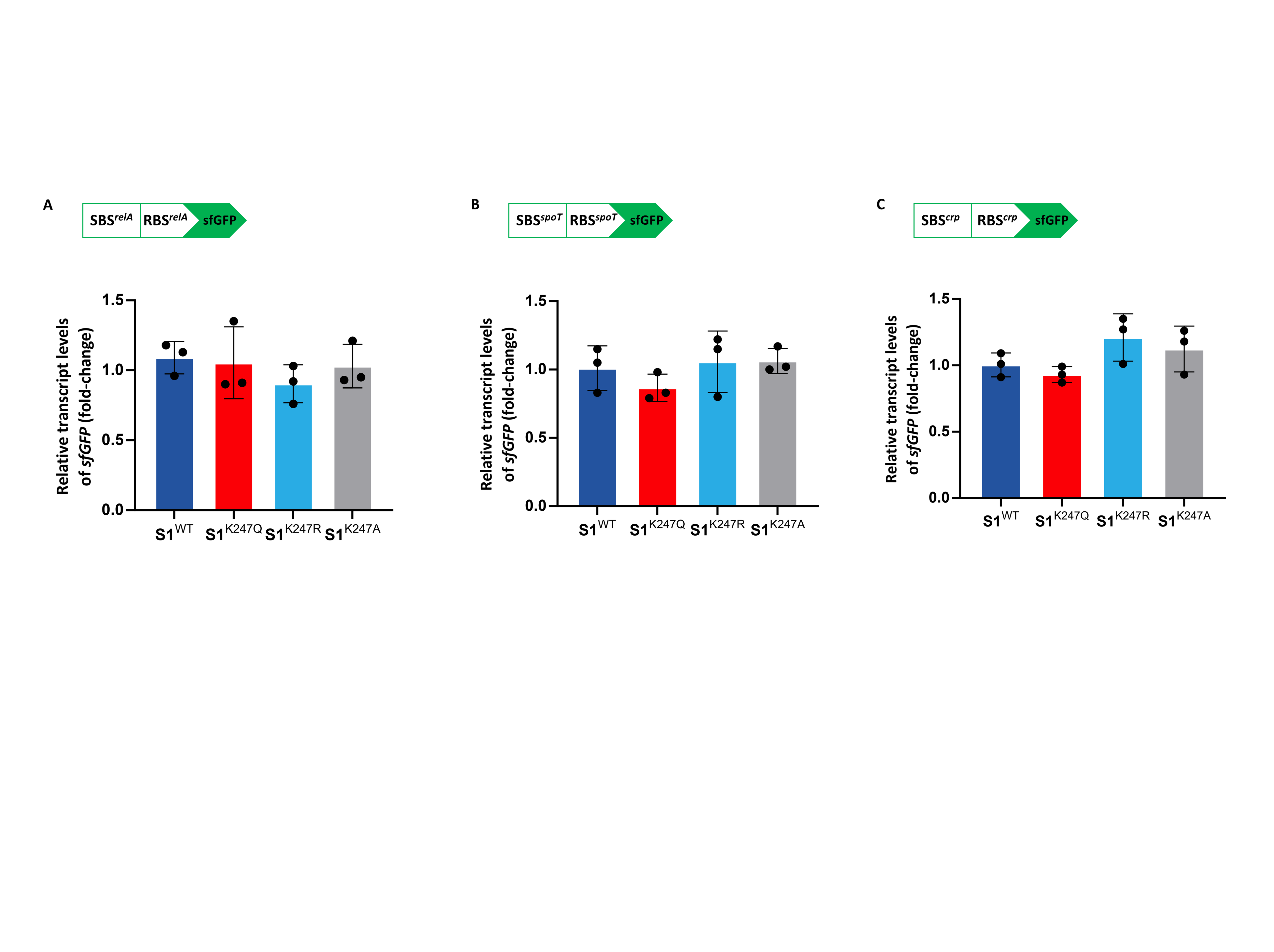


**Figure S2.** Transcription levels of *sfGFP* in the *E. coli* S1^WT^, S1^K247A^, S1^K247Q^ and S1^K247R^ strains that were engineered to express sfGFP. The strains were grown for 8 h in liquid LB medium at 37 °C. The mean value in the S1^WT^ strain was set to 1.0 (arbitrary units). The fold change represents the expression level compared to the mean value in the S1^WT^ strain.


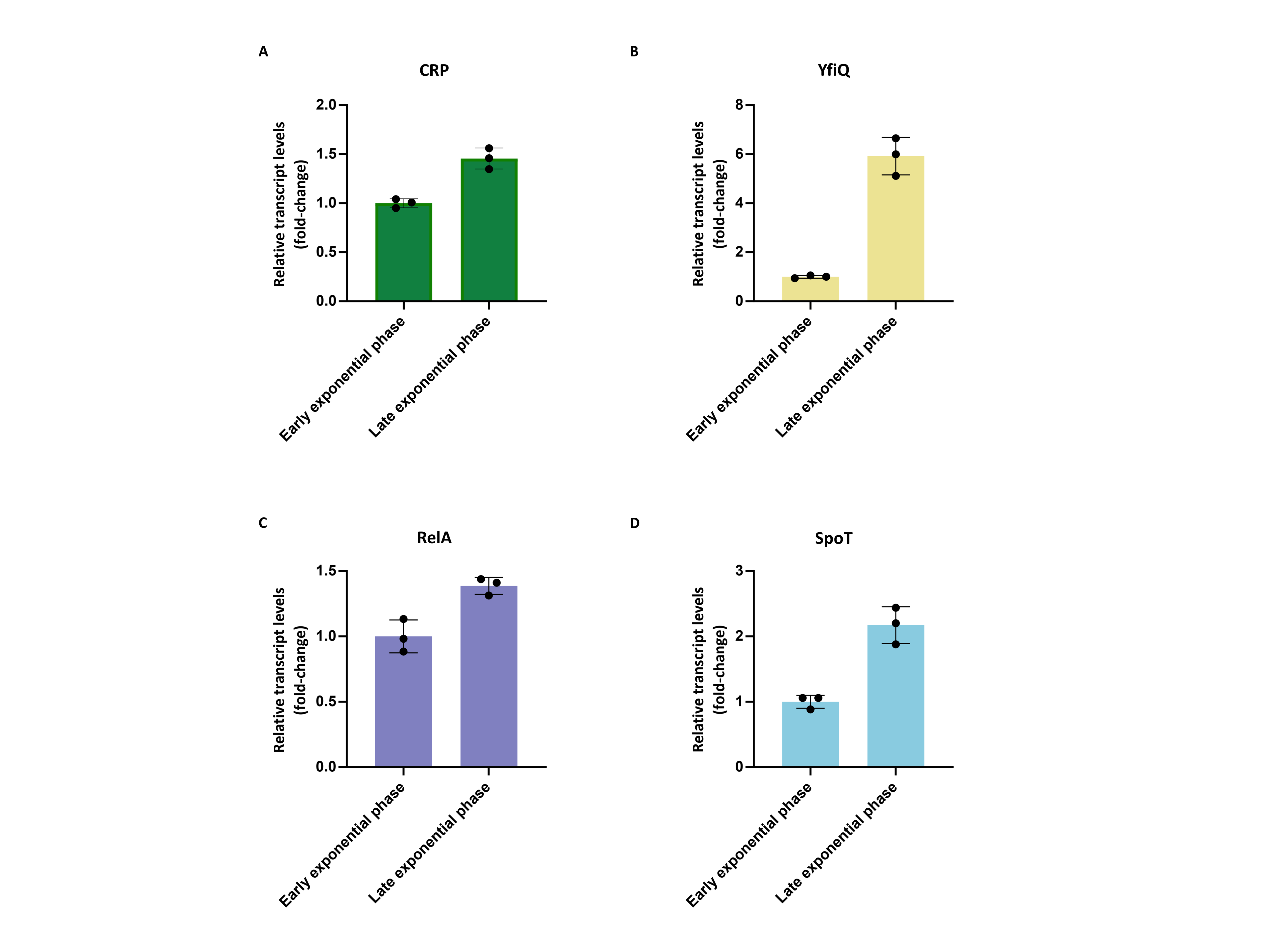


**Figure S3**. Transcription levels of *crp* (A), *yfiQ* (B), *relA* (C) and *spoT* (D) in *E. coli* strains grown in limited glucose (5 mM) at the early and late exponential phases. The fold change represents the expression level compared to that in the *E. coli* strains at the early exponential phase. The error bars show the SDs of three independent experiments, and the mean values from independent experiments were used. The mean value in the *E. coli* strains at the early exponential phase was set to 1.0 (arbitrary units).

**Table S1 Strains and plasmids used in this work**

| **Strain or plasmid** | **Source or Reference** |
| --- | --- |
| **Strains** |  |
| *E. coli* MG1655(WT) | NCBI: txid511145 |
| *E. coli* DH5α | Transgen Biotech |
| *E. coli* BL21(DE3) | Transgen Biotech |
| *E. coli* BL21(DE3)::pProEX-*crp* | This study |
| Δ*crp* | This study |
| *Ccrp* | This study |
| Δ*crp::relA* | This study |
| Δ*crp::spoT* | This study |
| Δ*crp::relA::spoT* | This study |
| *ΔrpsA::*S1^WT^ | This study |
| Δ*rpsA::*S1^K247Q^ | This study |
| Δ*rpsA::*S1^K247R^ | This study |
| Δ*rpsA::*S1^K247A^ | This study |
| Δ*rpsA::*S1^WT^*::*SBS*^relA^*-RBS*^relA^-sfGFP* | This study |
| Δ*rpsA::*S1^K247Q^*::*SBS*^relA^*-RBS*^relA^-sfGFP* | This study |
| Δ*rpsA::*S1^K247R^*::*SBS*^relA^*-RBS*^relA^-sfGFP* | This study |
| Δ*rpsA::*S1^K247A^*::*SBS*^relA^*-RBS*^relA^-sfGFP* | This study |
| Δ*rpsA::*S1^WT^*::*SBS*^spoT^*-RBS*^spoT^-sfGFP* | This study |
| Δ*rpsA::*S1^K247Q^*::*SBS*^spoT^*-RBS*^spoT^-sfGFP* | This study |
| Δ*rpsA::*S1^K247R^*::*SBS*^spoT^*-RBS*^spoT^-sfGFP* | This study |
| Δ*rpsA::*S1^K247A^*::*SBS*^spoT^*-RBS*^spoT^-sfGFP* | This study |
| Δ*rpsA::*S1^WT^*::*SBS*^crp^*-RBS*^crp^-sfGFP* | This study |
| Δ*rpsA::*S1^K247Q^*::*SBS*^crp^*-RBS*^crp^-sfGFP* | This study |
| Δ*rpsA*::S1^K247R^::SBS*^crp^*-RBS*^crp^-sfGFP* | This study |
| Δ*rpsA*::S1^K247A^::SBS*^crp^*-RBS*^crp^-sfGFP* | This study |
| **plasmid** |  |
| pPROEX-HTb | Thermo Scientific |
| pPROEX-HTb-*crp* | This study |
| pKD238*-*S1^WT^ | This study |
| pKD238*-*S1^K247Q^ | This study |
| pKD238*-*S1^K247R^ | This study |
| pKD238*-*S1^K247A^ | This study |
| pWT021a*-crp* | This study |
| pWT021a*-relA* | This study |
| pWT021a*-spoT* | This study |
| pWT021a*-relA-spoT* | This study |
| pUC19*-*SBS*^relA^*-RBS*^relA^-sfGFP* | This study |
| pUC19*-*SBS*^spoT^*-RBS*^spoT^-sfGFP* | This study |
| pUC19*-*SBS*^crp^*-RBS*^crp^-sfGFP* | This study |
| pET28a-T7-*RgTAL* | This study |
| pET28a-T7-CMDE-*RgTAL*  pR-T7-His*Pstrsts*2-*Sc4cl*-*Pcpal*1  pR-T7-CMDE-His*Pstrsts*2-*Sc4cl*-*Pcpal*1 | This study  This study  This study |

**Table S2 The oligonucleotides used in the study**

| Oligonucleotides | Sequence (5’to 3’) |
| --- | --- |
| **Primers for overproduction of CRP protein** | |
| ppro-*crp*-F | GGGCGCCATGGGATCCGGAATTATGGTGCTTGGCAAACCGCAAAC |
| ppro-*crp*-R | CTCATCCGCCAAAACAGCCAAGCTTACGAGTGCCGTAAACGACGA |
| ppro-thb-F | AAGCTTGGCTGTTTTGGCGGATGAG |
| ppro-thb-R | CATAATTCCGGATCCCATGGCGCCC |
| **Primers for construction of S1^WT^/S1^K247Q^****/S1^K247R^/S1^K247A^ gene in Δ*rpsA*** | |
| pkd238-S1-F | ATGGACTACAAGGACGACGATGACAAGATGACTGAATCTTTTGCTCA |
| pkd238-S1-R | TTTATTTGATGCCTGGTCTAGATTATTACTCGCCTTTAGCTGCTT |
| pkd238-F | TAATCTAGACCAGGCATCAAATAAAACGAAAGG |
| Pj23119-F | TCTAGAGTCACACAGGAAAGTACTAGATGGACTACAAGGACGACGA |
| Pj23119-R | CTTTCCTGTGTGACTCTAGAGCTAGCACAGTCCCTAGGAC |
| **Primers for Site-Directed Mutagenesis of S1** | |
| K247Q-F | ACTGTTAAAGTGCTGCAGTTCGACCG |
| K247Q-R | GCAGCACTTTAACAGTGATTTCGTC |
| K247R-F | CTGTTAAAGTGCTGAGGTTCGACCGC |
| K247R-R | CTCAGCACTTTAACAGTGATTTCGTC |
| K247A-F | ACTGTTAAAGTGCTGGCGTTCGACCGC |
| K247A-R | GCCAGCACTTTAACAGTGATTTCGTC |
| **Primers for EMSA** | |
| *relA*-F | AGCCAGTGGCGATAAGTTCCGGCGCTGGTGGAAAAA |
| *relA*-R | AGCCAGTGGCGATAAGACATATCCAGCATCGCCAGT |
| *spoT*-F | AGCCAGTGGCGATAAGCGGAGTCTGCGAGGACGC |
| *spoT*-R | AGCCAGTGGCGATAAGGCGTGCCTTGAGCCATGA |
| **Primers for real-time RT-PCR** | |
| RT-*crp*-F | CAGACCCGACTCTCGAATGG |
| RT-*crp*-R | AGCGTTTCCGCTTTTTCACC |
| RT-*spoT*-F | TGGGTTTTGAGGCGCTGTAT |
| RT-*spoT*-R | TCGAGTGAAAACGCTGCTCT |
| RT-*relA*-F | ATGCCAACGTAGTCAGCGAA |
| RT-*relA*-R | CGACCTGTTCGGAGGAAACA |
| 16s rRNA-F | TCGCGTTGCATCGAATTAAA |
| 16s rRNA-R | CCCCCTGGACGAAGACTGAC |
| RT-*yfiQ*-F | CGTCGATGAAACCCAATCGC |
| RT-*yfiQ*-R | GATCTTCGTGTTGCGATGCC |
| RT-*rpsA*-F | GACCAACAAAAACATCCACCC |
| RT-*rpsA*-R | GTCAACCTGCAGAACAACTGC |
| RT-*sfGFP*-F | GTTCACTGGTGTCGTCCCTA |
| RT-*sfGFP*-R | GCCAAGGTACCGGCAGTTTA |
| **Primers for construction of plasmids for assessing gene translation levels** | |
| pUC19-F | GGATCCCCGGGTACCGAGCTCGAAT |
| pUC19-R | AAGCTTGGCGTAATCATGGTCATAG |
| *relA*-*sf*GFP-F1 | CTATGACCATGATTACGCCAAGCTTAATGTGTGGAATTGATTTCGGCAGGTCTGGTCCC |
| *relA*-*sf*GFP-F2 | ATTTCGGCAGGTCTGGTCCCTAAAGGAGAGGACGATGCGTAAAGGCGAAGAGCT |
| *sfGFP*-R0 | ATTCGAGCTCGGTACCCGGGGATCCTCATTTGTACAGTTCATCCAAAC |
| *crp*-*sfGFP*-F1 | CTATGACCATGATTACGCCAAGCTTAATGTGTGGAATTCTCTGGAGAAAGCTTATAACA |
| *crp*-*sfGFP*-F2 | TCTGGAGAAAGCTTATAACAGAGGATAACCGCGCATGCGTAAAGGCGAAGAGCT |
| *spoT*-*sfGFP*-F1 | CTATGACCATGATTACGCCAAGCTTAATGTGTGGAATTCTGAAGGTCGTCGTTAATCAC |
| s*poT*-*sfGFP*-F2 | TGAAGGTCGTCGTTAATCACAAAGCGGGTCGCCCATGCGTAAAGGCGAAGAGCT |
| **Primers for overexpression of *relA*/*spoT*****/*relA-spoT*/*crp in* Δ*crp*** | |
| pWT-F | ATTGCGTTGCGCACTTAATTAACGG |
| pWT-R | GGGTACCTTTCTCCTCTTTAATGAATTCGCT |
| *spoT*-F | TTCATTAAAGAGGAGAAAGGTACCCTTGTATCTGTTTGAAAGCCT |
| *spoT*-R | CCGTTAATTAAGTGCGCAACGCAATTTAATTTCGGTTTCGGGTGA |
| *relA*-F | TTCATTAAAGAGGAGAAAGGTACCCATGGTTGCGGTAAGAAGTGC |
| *relA*-R | CCGTTAATTAAGTGCGCAACGCAATCTAACTCCCGTGCAACCGAC |
| *relA*-*spoT*-F | CGCGCGTCGGTTGCACGGGAGTTAGTTTACGGCTAGCTCAGTCCT |
| *relA*-*spoT*-R | CTAACTCCCGTGCAACCGACGCGCG |
| *crp*-F | TTCATTAAAGAGGAGAAAGGTACCCATGGTGCTTGGCAAACCGCA |
| *crp*-R | CCGTTAATTAAGTGCGCAACGCAATTTAACGAGTGCCGTAAACGA |
| SmR-R0 | CAAATAAACGCCATGGGCAT |
| pWT-F0 | TCATTTTCGCCAGATATCGACGTC |
| **Primers for creation and identification mutant Δ*crp*** | |
| *crp*-ko-F1 | TCCAGAGACAGCGGCGTTATCTGGCTCTGGAGAAAGCTTATAACAGAGGATAACCGCGC |
| *crp*-ko-R1 | GCGCCACATCGGGGGAAACAAAATGGCGCGCTACCAGGTAACGCGCCACTCCGACGGGA |
| *crp*-*kanR*-F | ATAACAGAGGATAACCGCGCATGAGCCATATTCAACGGGA |
| *crp*-*kanR*-R | AACGCGCCACTCCGACGGGATTAGAAAAACTCATCGAGCA |
| *crp*-ko-F | GACACAAAGCGAAAGCTATGC |
| *crp*-ko-R | CCGAATCGTAATTCGCCAAG |
| **Primers for construction of plasmids for product synthesis** | |
| pET28a-F | GAAAGGAAGCTGAGTTGGCT |
| pET28a-R | AGCTCTTCGCCTTTACGCATGTGGTGGTGGTGGTGGTGGG |
| CMDE-F | ATGGGCAACCGGATGCATAA |
| CMDE-R | TGATGATGGCTGCTGCCCATCGTCCTCTCCTTTAGGGACC |
| pET28a-F1 | ATGGGCAGCAGCCATCATCA |
| pET28a-R1 | TTATGCATCCGGTTGCCCATGGTATATCTCCTTCTTAAAG |
| CMDE-F1 | TTTAACTTTAATAAGGAGATATACCATGGGCAACCGGATGCATAA |
| CMDE-R1 | CGTCCTCTCCTTTAGGGACCAGACCTGCCGAAATCGAGTATCTTTCGTATGTGAT |
| pET28a-F2 | GGTCTGGTCCCTAAAGGAGAGGACGATATACCATGGGCAGCAGCCATCACCA |
| pET28a-R2 | GGTATATCTCCTTATTAAAGTTAAACAAAATTATTTCTACAGG |
| *sfGFP*-F | ATGCGTAAAGGCGAAGAGCT |
| *sfGFP*-R | AGCCAACTCAGCTTCCTTTCTTTGTACAGTTCATCCAAAC |
